# The *MTHFR* C677T Variant is Associated with Responsiveness to Disulfiram Treatment for Cocaine Dependency

**DOI:** 10.3389/fpsyt.2012.00109

**Published:** 2013-01-14

**Authors:** Catherine J. Spellicy, Thomas R. Kosten, Sara C. Hamon, Mark J. Harding, David A. Nielsen

**Affiliations:** ^1^Menninger Department of Psychiatry and Behavioral Sciences, Baylor College of Medicine and Michael E. DeBakey V. A. Medical CenterHouston, TX, USA; ^2^Laboratory of Statistical Genetics, The Rockefeller UniversityNew York, NY, USA

**Keywords:** genes, disulfiram, polymorphism, cocaine, treatment, dependence

## Abstract

**Objective:** Disulfiram is a one of the few pharmacotherapies for cocaine addiction that shows promise. Since disulfiram and cocaine both affect levels of global methylation we hypothesized the *MTHFR* gene, whose product is involved in supplying methyl groups for DNA and protein methylation, may be associated with responsiveness to disulfiram in cocaine-dependent individuals. **Methods:** Sixty-seven cocaine-dependent patients were stabilized on methadone for 2 weeks and then randomized into disulfiram (250 mg/day, *N* = 32) and placebo groups (*N* = 35) for 10 weeks. Patients were genotyped for the *MTHFR* (rs1801133, also known as C677T) polymorphism and the data was evaluated for association with cocaine-free urines in the disulfiram or placebo groups. Data from patients that completed all 10 weeks of the study (*N* = 56) were analyzed using repeated measures analysis of variance (ANOVA), corrected for population structure. **Results:** The CT or TT *MTHFR* genotype group (*N* = 32) dropped from 73 to 52% cocaine-positive urines on disulfiram (*p* = 0.0001), while the placebo group showed no treatment effect. The CC *MTHFR* genotype group (*N* = 24) showed a smaller, but still significant, reduction in cocaine-positive urines on disulfiram compared to placebo; 81–69% (*p* = 0.007). **Conclusion:** This study indicates that a patient’s *MTHFR* genotype may be used to identify individuals who might show improved response to disulfiram treatment for cocaine dependence. **Clinical Trial:** Pharmacogenetics of Disulfiram for Cocaine, clinicaltrials.gov/ct2/show/NCT00149630, NIDA-18197-2, NCT00149630.

## Introduction

Substance abuse is a critical problem in the United States. Cocaine is one of the most common drugs of abuse and the effects of cocaine dependency (CD) lead to social and physiological morbidities. Drug abuse and drug addiction costs employers over 122 billion dollars in lost productivity and time, and another 15 billion dollars in health insurance costs each year [Substance Abuse and Mental Health Services Administration (SAMHSA), 2011] indicating that substance abuse and addiction have significant economic impact. CD also may lead to risky behaviors making HIV, illicit opioid use, and emergency care more common [Substance Abuse and Mental Health Services Administration (SAMHSA), 2011].

Although CD is currently without an FDA-approved pharmacotherapy, some drugs show promise in treatment of CD, an example of which is disulfiram (Carroll, 1993; Carroll et al., 1998, 2004; George et al., 2000; Petrakis et al., 2000). Disulfiram, also known as Antabuse, is used to treat alcoholism via its blockage of the conversion of acetaldehyde (a metabolite of ethanol) into acetic acid, thereby inducing aversive physiological symptoms such as nausea and vomiting. The exact mechanism by which disulfiram exerts its effect to reduce cocaine cravings and/or use is not exactly known. However, studies have shown that genetic background may be important in determining responsiveness to disulfiram as a CD pharmacotherapy (Kosten et al., 2012).

CD is a complex disease with a strong genetic component as indicated by a heritability of up to 72% (Tsuang et al., 1998; Goldman et al., 2005) and increased odds ratios up to 40.8 in monozygotic twins as compared to dizygotic twins (Kendler and Prescott, 1998). Studies have shown that individual genetic variants are associated with risk of substance abuse, including CD [but usually only in select populations, e.g., *OPRM1* (Berrettini et al., 1997), *Homer1* (Dahl et al., 2005), *OPRD1* (Kreek et al., 2005; Crist et al., 2012)] but also that epigenetics may play a significant role in the disease process (e.g., Nielsen et al., 2009, 2010). Changes in epigenetics, including chromatin structure and DNA methylation, due to drug use, and the resulting change in gene expression are hypothesized to contribute to the neural plasticity in drug users that has long been considered a molecular mechanism through which drug addiction and relapse may occur (e.g., Berke et al., 1998; Nestler, 2001; Grimm et al., 2003; Yao et al., 2004; Shaham and Hope, 2005; Kalivas and O’Brien, 2008; Wong et al., 2011). Cocaine itself is known to affect gene expression via regulating heterochromatin (Maze et al., 2011) and other epigenetic factors (Nielsen et al., 2012b).

Disulfiram, although not a drug of abuse, also falls into this category of pharmacotherapies that may modify the epigenetic landscape. Disulfiram causes hypomethylation in tumor cell lines via its inhibition of DNA methyltransferase 1 (DNMT1; Lin et al., 2011). DNMT1 is an enzyme involved in maintenance of DNA methylation patterns following mitosis (Kinney and Pradhan, 2011).

**Figure 1:**
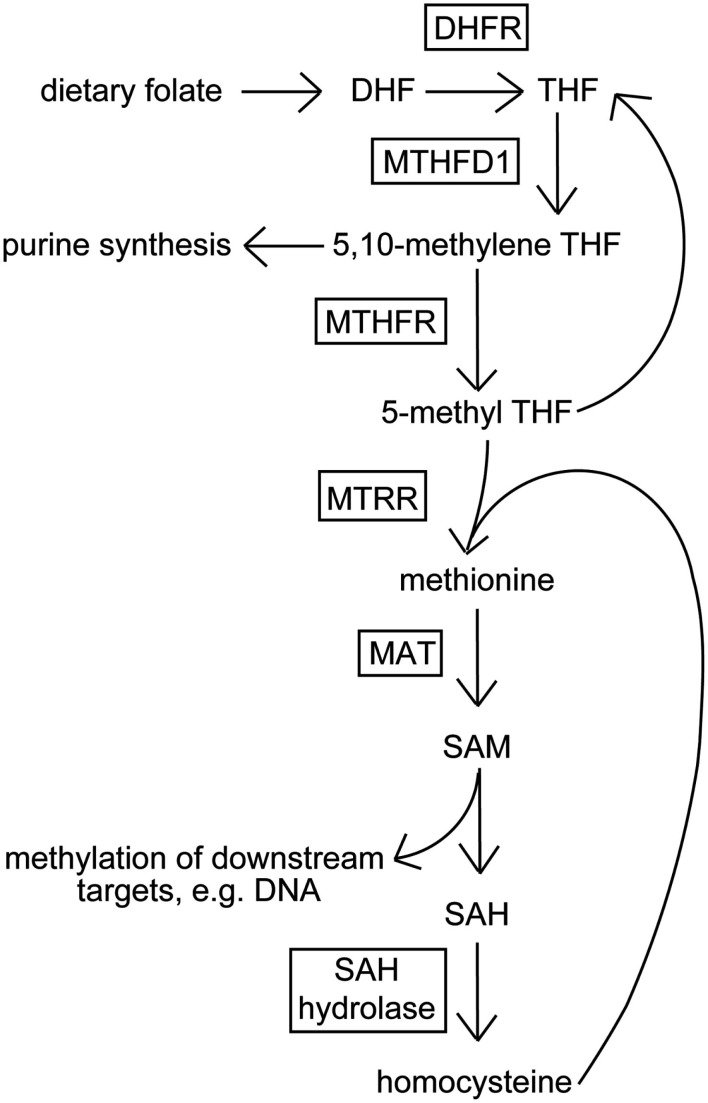
**Schematic of the folic acid metabolic cycle**. Dietary folate (vitamin B12) is converted to dihydrofolate (DHF), and subsequently metabolized to tetrahydrofolate (THF) by dihydrofolate reductase (DHFR). THF is converted to 5,10-methylene THF by methylenetetrahydrofolate dehydrogenase 1 (MTHFD1), then to 5-methyl THF by methylene tetrahydrofolate reductase (MTHFR). 5-methyl THF is demethylated and combined with homocysteine to form methionine by 5-methyltetrahydrofolate-homocysteine methyltransferase reductase (MTRR). Methionine is transformed to S-adenosyl methionine (SAM) by methionine S-adenosyltransferase (MAT). SAM-dependent methyltransferases metabolize SAM into S-adenysyl homocysteine (SAH) methylating downstream targets. SAH is converted to homocysteine by SAH hydrolase.

In this study, we chose to examine the relationship between response to disulfiram (in terms of cocaine-positive urines over time) and a single nucleotide polymorphism (SNP, rs1801133, also known as C677T) in the gene coding for 5,10-methylene tetrahydrofolate reductase (MTHFR), whose product is central to the folic acid metabolic cycle (see Figure 1). The folate cycle is critical to normal cellular function via supplying metabolites used for nucleotide and amino acid synthesis, and for methylation of downstream DNA and proteins. The minor T allele of rs1801133 codes for an amino acid change that leads to a more thermolabile enzyme *in vitro* (Frosst et al., 1995), and has also been found to associate with neural tube defects (NTDS) such as spina bifida, a condition in which the neural tube, the precursor to the central nervous system (CNS) during embryonic development, fails to close completely leaving neural tissue open to the environment (Whitehead et al., 1995; Harisha et al., 2010).

Given its involvement in both development and function of the CNS, and its importance in biochemical pathways, such as nucleotide synthesis and methylation, we hypothesized that variants in *MTHFR* may be associated with response to disulfiram treatment in cocaine-dependent individuals.

## Materials and Methods

### Participants

Ninety-three cocaine- and opioid co-dependent patients were enrolled, consented, and stabilized on methadone treatment during a 2-week screening period at Yale University (*N* = 40) and Baylor College of Medicine (*N* = 53). The final number of patients with at least one cocaine-positive urine sample, and that were able to be genotyped, was 67. Opioid and/or cocaine dependence was diagnosed via interview and the DSM-IV criteria. The MINI (English Version 5.0.0, July 1, 2006; Sheehan et al., 1998) and the ASI (McLellan et al., 1992) were performed on all participants to obtain baseline psychiatric characteristics. Patients were excluded based on current diagnosis of other drug or alcohol dependence (excluding tobacco), current major medical illness not stabilized on medications, a history of major psychiatric disorder (psychosis, schizophrenia, bipolar), current suicidality, or an inability to read and understand the consent form. Women of childbearing age required a negative urine pregnancy test, use of adequate contraception to prevent pregnancy during the study, and monthly pregnancy tests. All participants signed an informed consent approved by Yale University and the Baylor College of Medicine Institutional Review Boards that gave specific consent for genetic studies. Determination of ethnicity was based on self-report of ethnic/cultural background of the patients.

This was a 12 week study in which individuals were assigned randomly to placebo or disulfiram groups (250 mg daily). Methadone dose increased 5 mg/day from an initial 25 mg to a maintenance dose of 60 mg/day. Individual cognitive behavioral therapy was provided weekly to all patients (Carroll, 1997).

Supervised urine samples were obtained three times weekly and tested for the presence of a cocaine metabolite, benzoylecgonine, using an Olympus AU 640 Emit system (Olympus America Inc., Melville, NY, USA). The threshold for a positive urine concentration was 300 ng/mL. Saliva samples were collected for genotyping.

### Genotyping

DNAs were purified and genotyped as previously described (Kosten et al., 2012). Briefly, 10 mL Scope mouthwash was used to rinse the subject’s mouth for 60 s and centrifuged to obtain buccal cells. The Gentra Puregene Buccal Cell Kit (Qiagen, Valencia, CA, USA) was used to purify the DNAs following the manufacturer’s recommendations.

The *MTHFR* C/T rs1801133 genetic variant was genotyped using the TaqMan^®^ (Applied Biosystems, Foster City, CA, USA) primer-probe set (Assay ID C_1202883_20). PCR amplifications were performed using Platinum^®^ quantitative PCR SuperMix-UDG (Invitrogen, Carlsbad, CA, USA) on a GeneAmp^®^ PCR system 9700 and read on an ABI Prism 7900 detection system. SDS 2.2 software (Applied Biosystems, Foster City CA, USA) was used to analyze the results. Sex was determined via the presence of a sex-specific variant, rs11575897 in the *SRY* gene (C_32310143_10, Applied Biosystems). Ten ancestry-informative markers were evaluated to determine ethnicity (Lao et al., 2006). All genotyping experiments were performed in duplicate.

### Statistical analysis

Statistical analysis was performed as previously described (Kosten et al., 2012). Demographic data was analyzed using chi-squared or *t*-test. R version 2.9.1 (R Development Core Team, 2009) was used to perform a repeated measures analysis of variance (ANOVA) on the cocaine-positive urine data over time in the placebo/disulfiram groups, and by *MTHFR* genotype. We performed statistical comparisons between conditions (disulfiram or placebo), *MTHFR* genotypes (0 = CC genotype, 1 = all other genotypes), time (each 2 week period), and then examined potential interactions between condition and time, and/or between condition and genotype. We analyzed those individuals who had complete urine toxicology data (*N* = 56), and as a secondary analysis, included those individuals with incomplete urine toxicology data (*N* = 67). We calculated effect size as a partial eta-squared statistic using condition or SNP variance over residual variance. The three general cutoffs for effect size are the following: a small effect is 0.01, medium effect is 0.06, and large effect is 0.14.

To determine population structure, our cohort was compared against Centre d’Etude du Polymorphisme Humain – Human Genome Diversity Project (CEPH-HGDP) samples (1,035 subjects of 51 populations; Kosten et al., 2012) using the STRUCTURE 2.3.3 software (Pritchard et al., 2000; Hubisz et al., 2009).

## Results

### Baseline characteristics by treatment and *MTHFR* genetics

Sixty-seven patients, 32 that had been randomized to disulfiram treatment group and 35 to placebo group, were genotyped. The patients with complete urine toxicology data for the 12 weeks of the trial included 32 individuals with a CT or TT genotype and 24 individuals with a CC genotype. The patients had a mean age of 38 years and were mostly Caucasian (76%) and mostly male (71%). Twenty-eight (50%) patients had previously been in a methadone maintenance treatment program. On average, participants used cocaine for 12 years and for 17 days in the month prior to entering this study (see Table 1). An additional 11 patients were genotyped that had an incomplete urine toxicology and for whom demographic data is not shown.

**Table 1:** **Clinical and demographic characterization by treatment and genotype**.

*MTHFR* genotype	Treatment			
	Disulfiram		Placebo	
	CT/TT	CC	CT/TT	CC
*N*	14	10	18	14
% Male	64	70	72	61
% Caucasian	93	70	89	43
% African American	0	20	6	43
% Hispanic	7	10	6	14
% Employed	69	80	67	50
Age years (SD)	36 (8.1)	42 (10.9)	37 (9.1)	43 (13.1)
Cocaine last 30 days (SD)	15 (9.4)	14 (10.9)	19 (9.2)	21(9.1)
Cocaine years (SD)	6 (4.8)	13 (8.3)	14 (9.3)	14(8.5)
% Alcohol use	36	40	39	36
% Marijuana use	57	50	44	50
% Past methadone	29	70	56	57

*No significant baseline differences among the two treatment groups by genotype in any clinical characteristics after adjusting for multiple testing (*p* > 0.05)*.

Twenty-eight patients (50%) reported any alcohol use history (>1 year), and 21 patients (37.5%) reported marijuana use history. We found no significant baseline differences among the four treatment by genotype groups in any clinical characteristics except for cocaine use in years (“cocaine years”), but this became non-significant after adjusting for multiple testing (*p* > 0.05).

When the complete urine toxicology sample (*N* = 56) was compared to total sample (*N* = 67), there were no significant differences between the two samples for any of the demographic variables (Table 1). Additionally, when comparing demographic data for the 11 individuals excluded from analysis to the individuals included in the final analyses (*N* = 56), there were no significant differences for any of the demographic variables (*p* ≥ 0.1).

### Cocaine treatment outcomes by group

**Figure 2:**
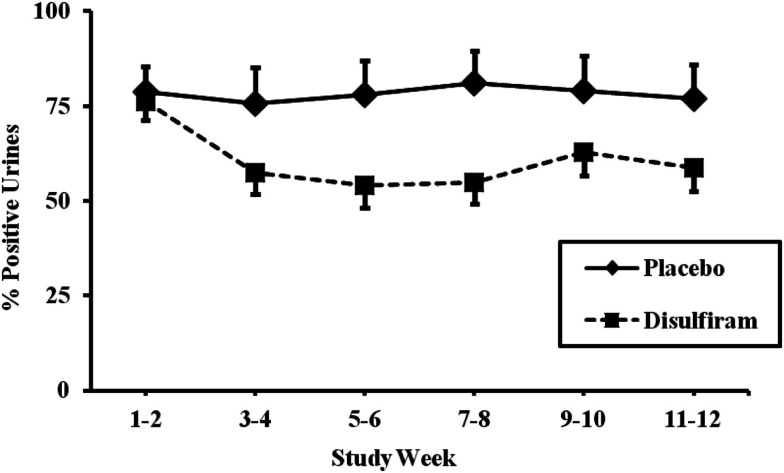
**Response to disulfiram therapy by treatment group (*N* = 56)**. Cocaine-positive urine toxicology screens are shown for each 2 week time period across the 12-week trial as percent positive urines. The placebo treatment group (*N* = 32) is represented by a solid line and the disulfiram (250 mg/day) treatment group (*N* = 24) is represented by a dashed line. Standard error bars are shown for each time point. Only data from study participants with complete urine toxicologies were included in the analysis.

We divided the 67 patients who had been genotyped for the *MTHFR* rs1801133 variant into two groups based on presence of the T (minor) allele. For the first analysis, only those individuals with complete urine toxicology for the 12 week study were included (*N* = 56). We first looked at all individuals in the placebo group as compared to the disulfiram group, regardless of genotype. Results showed that individuals in the placebo group did not have a significant reduction in cocaine-positive urines over time (79% positive at week 1 versus 77% positive at week 12), while individuals in the disulfiram group had a significant reduction in cocaine-positive urines over time (76% positive urines at week 1 versus 59% positive urines at week 12, *F* = 22.38; *d*f = 1, 332; *p* < 0.0001, with an effect size of 0.065; see Figure 2).

### Cocaine treatment outcomes by *MTHFR* genotype

**Figure 3:**
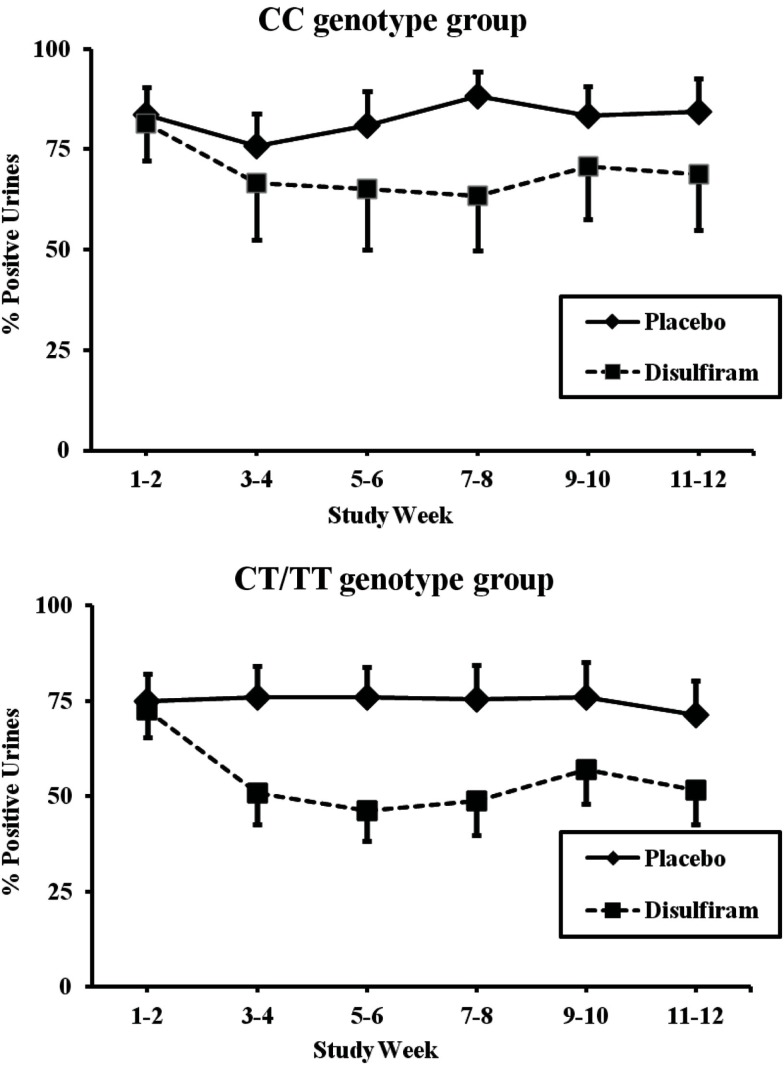
**Response of *MTHFR* C677T CC and CT/TT genotype groups to disulfiram pharmacotherapy in individuals with complete urine toxicology data (*N* = 56)**. Cocaine-positive urine toxicologies are shown for each 2 week time period across the 12-week trial in percent positive urines. The solid line represents time points for the placebo treatment group and the dashed line represents time points for the disulfiram treatment group (250 mg/day). Top panel: patients with the *MTHFR* CT/TT or TT genotype (disulfiram, *N* = 14; placebo, *N* = 18). Bottom panel: patients with *MTHFR* CC genotype (disulfiram, *N* = 10; placebo, *N* = 14). Standard error bars are shown for each time point.

The T allele carrier group, which included individuals with CT or TT *MTHFR* genotypes, showed better response to disulfiram than did individuals not carrying the rs1801133 minor T allele (*F* = 15.97; *d*f = 1, 182; *p* < 0.0001, and an effect size of 0.0806). The CC genotype group showed a more modest, but still significant, difference between disulfiram and placebo (*F* = 7.27; *d*f = 1, 134; *p* = 0.007, with an effect size of 0.0514). As shown in Figure 3, cocaine-positive urines for the T allele patients during the two baseline weeks were approximately 74% for both disulfiram and placebo treatment groups. These rates dropped to 52% during the last 2 weeks of treatment for the disulfiram group and to 71% for the placebo group (*F* = 6.982, *d*f = 1,134, *p* = 0.009 with an effect size of 0.0495). In comparison, baseline cocaine urines for the CC genotype group were approximately 83% for disulfiram and placebo treatment groups (see Figure 3). These rates decreased to 69% during the last 2 weeks for the disulfiram treatment group and were unchanged for the placebo group.

When all individuals were analyzed together, which included those with missing data points, the results were similar. Without regard to genotype, the placebo group had a non-significant reduction in cocaine-positive urines from 80 to 75%, while the disulfiram group had significant reduction in cocaine-positive urines from 79 to 62% (*F* = 16.44, *d*f = 1, 358; *p* < 0.0001). When *MTHFR* rs1801133 genotype was considered in the analysis, the T allele genotype group, again, responded better to disulfiram. The T allele genotype group dropped from 79% cocaine-positive urines at baseline to 52% positive urines at 12 weeks (*F* = 11.01; *d*f = 1, 198; *p* = 0.001), while the CC genotype group showed a less dramatic reduction from 81% positive urines to 69% (*F* = 6.36; *d*f = 1, 154; *p* = 0.0127).

## Discussion

In this study, we tested the hypothesis that the C677T (rs1801133) variant in *MTHFR* is associated with response to disulfiram treatment in CD individuals. Our data support the hypothesis that individuals who had at least one copy of the minor *MTHFR* (T) rs1801133 allele showed better response to disulfiram than those individuals without a T allele.

The SNP examined in this study, rs1801133 or C677T, is a non-synonymous transition known to alter the enzyme activity of MTHFR. The 677T allele codes for an alanine to valine amino acid change and produces a thermolabile form of the enzyme that has 50–60% reduced enzyme activity at increased temperatures than the protein resulting from the allele with the more common 677C variant (Frosst et al., 1995). As mentioned previously, *MTHFR* is a key component of the folic acid metabolic cycle, and is therefore central to maintaining the balance of folic acid (folate, pteroylglutamic acid) to homocysteine in the body. Typically an inverse relationship exists; as folate increases, homocysteine decreases. Higher systemic levels of homocysteine (i.e., lower levels of folate) correlate with decreased cognitive function, and are associated with white matter lesions in the brain (de Lau et al., 2007, 2009). Higher systemic levels of folate (i.e., lower levels of homocysteine) have been found to be associated with increased cognitive function (Morris et al., 2001; Malouf and Grimley Evans, 2008; Moretti et al., 2008; Sen and Kanani, 2009; Tangney et al., 2009). For example, increased folate levels in elderly individuals were found to be associated with slower cognitive decline, while increased homocysteine levels were associated with faster cognitive decline (Tangney et al., 2009). Another study illustrated that elderly individuals who took folic acid supplements daily showed significant improvement in global cognitive functioning (Malouf and Grimley Evans, 2008) and a similar effect was observed in school-aged girls supplemented with both folate and iron (Sen and Kanani, 2009). Likewise, folate deficiency in the CNS causes cognitive deficits such as dyskinesia, psychomotor retardation, intellectual delay (Moretti et al., 2008), and low folate levels were found to be associated with reduced memory function (Morris et al., 2001).

5,10-Methylene tetrahydrofolate reductase is known to be a critical player in the development of the CNS in humans. Low folate levels are associated with increased incidence of NTDs (van der Linden et al., 2006; Greene et al., 2009; Harris, 2009), a medical condition in which the neural tube, the developmental precursor to the CNS, does not close fully and leaves neural tissue open to the environment. Folic acid supplementation was so successful in reducing the occurrence and recurrence of NTDs that mandatory folate fortification of grain products was initiated by the FDA in 1998 (MRC Vitamin Study Research Group, 1991; Czeizel and Dudas, 1992; Food and Drug Administration, 1996; Berry et al., 1999).

Individuals homozygous for the low activity T allele of *MTHFR* have higher overall plasma levels of homocysteine (Jacques et al., 1996; Rozen, 1997; Yang et al., 2008). The 677T variant has been associated with various psychological conditions including depression and/or loneliness, and with attention deficit hyperactivity disorder in specific patient populations (Hickie et al., 2001; Krull et al., 2008; Holmes et al., 2011; Kamdar et al., 2011; Lan et al., 2012).

Because MTHFR and folic acid metabolism are important for providing metabolites for methylation, and because disulfiram and cocaine can both affect DNA and/or histone methylation levels, the pharmacogenetic effect in disulfiram treatment for CD may be related to the interaction of these epigenetic effects. The T allele of *MTHFR* variant rs1801133 has been found to be associated with higher homocysteine levels (Jacques et al., 1996; Rozen, 1997; Yang et al., 2008), and increased homocysteine levels (and its metabolites) have been found to be associated with global DNA hypomethylation (Yi et al., 2000; Caudill et al., 2001). In addition, hypomethylation was observed in women carrying one minor T allele in conjunction with folate deficiency, and in women carrying two minor T alleles of *MTHFR* rs1801133 in the presence of vitamin B6 deficiency (Axume et al., 2007; La Merrill et al., 2012). Similarly, mice with one or both copies of *MTHFR* knocked out showed hyperhomocysteinemia as well as global DNA hypomethylation (Chen et al., 2001). Cocaine itself can affect epigenetics either at the DNA or histone level. For example, acute cocaine administration in mice increases local methylation of specific genes via upregulating DNA methyltransferases in the brain (Anier et al., 2010). In contrast, chronic cocaine administration or use decreased H3K9 histone methylation (Maze et al., 2010; Covington et al., 2011), decreased global DNA methylation (in offspring of mice who had been administered chronic cocaine; Novikova et al., 2008), and decreased heterochromatinization (Maze et al., 2011). Disulfiram also can affect the epigenetic landscape by decreasing global DNA methylation (Lin et al., 2011). Methylation is an important process by which gene expression is mediated, and may be the basis of the genetic effect observed in disulfiram treatment for CD and *MTHFR* genotype. Additional research is required to test this hypothesis and to further elucidate the specific underpinnings of the effect of *MTHFR* alleles on CD pharmacotherapies such as disulfiram.

We have recently published two studies showing associations between response to disulfiram with the genes coding for dopamine-β hydroxylase (*D*β*H*; Kosten et al., 2012), the serotonin transporter (*5-HTTLPR*), and tryptophan hydroxylase two (*TPH2*; Nielsen et al., 2012b). These studies also illustrated a pharmacogenetic effect in disulfiram therapy. Specifically, the first study found that the group homozygous for the major C allele of *D*β*H* showed a treatment response with disulfiram (e.g., decreased cocaine-positive urines over the 12-week trial), while the minor T allele, which associates with lower DβH levels, did not. Nielsen et al. (2012b), showed that subjects with at least one copy of the minor allele of *5-HTTLPR* or *TPH2*, or both, responded to disulfiram therapy, while the genotype groups with the major alleles of both genes did not show reduced cocaine-positive urines (Nielsen et al., 2012a). With the addition of the new findings in this study, that the C677T variant in the folate metabolism gene *MTHFR* is a potential modifying factor in disulfiram pharmacotherapy for cocaine dependence, we propose that the genetic profile of an individual including findings such as these may be used to help in tailoring a more effective pharmacotherapy for CD.

In conclusion, this study identifies the C677T variant in the folate metabolism gene, *MTHFR*, as a potential modifying factor in disulfiram pharmacotherapy for cocaine dependence. Specifically, individuals carrying the minor T allele showed increased response to disulfiram treatment. This finding may be useful in tailoring treatment for CD to an individual based on genotype, thereby increasing the efficacy of CD pharmacotherapy. However, this is a clinical trial with a small cohort therefore replication studies will be required to confirm these results.

## Conflict of Interest Statement

The authors declare that the research was conducted in the absence of any commercial or financial relationships that could be construed as a potential conflict of interest.
